# Cigarette sharing and gifting in China: Patterns, associated factors, and behavioral outcomes

**DOI:** 10.18332/tid/144054

**Published:** 2022-01-28

**Authors:** Dan Wu, Guihua Jiao, Huan Hu, Lu Zhang, Lixin Huang, Shuhan Jiang

**Affiliations:** 1 School of Psychology, Center for Mental Health, Shenzhen University, Shenzhen, China; 2 Department of Psychology, Research Center on Quality of Life and Applied Psychology, Guangdong Medical University, Dongguan, China; 3 School of Humanities and Management, Zhejiang Chinese Medical University, Hangzhou, China

**Keywords:** cigarette sharing, cigarette gifting, beliefs, social participation, behavioral outcome

## Abstract

**INTRODUCTION:**

The purpose of this study was to investigate the patterns and factors associated with cigarette sharing and gifting, and to explore whether smoking can be predicted by these social practices.

**METHODS:**

A cross-sectional survey using a multi-stage sampling design was conducted online from 30 April to 30 July 2020 in China. A sample of 982 household heads from Guangdong Province and 530 household heads from Shaanxi province were involved in the data analysis. Demographic characteristics, social participation, beliefs and behaviors related to cigarette sharing and gifting were assessed. Chi-squared analysis and multiple logistic regression analysis were used to explore the key factors associated with cigarette sharing and gifting, and to identify their relationship with smoking.

**RESULTS:**

The shared and gift cigarettes were both mainly offered to friends, and receiving gift cigarettes mostly occurred during the holidays. Gender and province were associated with cigarette sharing, and marital status and social participation were associated with cigarette gifting. Cigarette gifting beliefs and smoking status were prominent predictors for both sharing and gifting cigarettes. Cigarette gifting beliefs were significantly higher among smokers than non-smokers, and people with high cigarette gifting beliefs were 1.68 (adjusted odds ratio, AOR=19.17; 95% CI: 13.31–27.61) times more likely to be a smoker. Offering shared cigarettes has been found to significantly predict tobacco use (AOR=19.17; 95% CI: 13.31–27.61), while people who received shared and gift cigarettes were 1.50 (95% CI: 1.08–2.09) and 2.58 (95% CI: 1.66–4.00) times more likely to be a current smoker than those who did not receive cigarettes, respectively.

**CONCLUSIONS:**

Cigarette sharing and gifting were especially pervasive among male smokers and married people in Shaanxi Province. Offering shared cigarettes and receiving shared/gift cigarettes might facilitate cigarette use. This study provides evidence-based data to support the design and implementation of tobacco control programs for the denormalization of gifting and sharing cigarettes.

## INTRODUCTION

Globally in 2019, the overall number of global current smokers had reached 1.14 billion^1^. Smoking tobacco use accounted for 7.69 million deaths and 200 million disability-adjusted life-years in 2019, which was one of the leading risk factors for death, especially for males^1^. However, the irrational phenomenon of sharing and gifting cigarettes is still endemic.

As early as the late 15th century, tobacco was given as a gift from the Native Americans to Christopher Columbus, and soon introduced and spread in Europe^2^. Cigarettes were used as appropriate Christmas gifts in the United States from the 1930s to the 1960s^3^. Since cigarettes were introduced to China, this practice has become increasingly popular across the country. China has a long history of strong influence from Confucian culture, which includes respecting superiors, emphasizing social ties (guanxi), and valuing reciprocity via gift exchange to maintain harmonious social bonds in Chinese society^4,5^. Chinese tobacco manufacturers take full advantage of the cigarette gifting and sharing culture demonstrated by packs with imagery and terminology^6^. A recent online nationwide cross-sectional study showed most current smokers reported an experience of sharing (97%) and gifting (90%) cigarettes in China^7^. Understanding the determinants of cigarette sharing and gifting is an important issue for future tobacco control research. However, few empirical studies have been conducted to depict the status of cigarette sharing and gifting or examined the associated factors in China^8^.

Considering the potential hazardous effects, the assumption that cigarette sharing and gifting may promote smoking is plausibly effective through two pathways. Firstly, Health Belief Model and Cognitive Behavioral Theory both emphasize that beliefs are the key influencing factor for individual behaviors^9,10^. Willingness to receive smoking-related gifts was associated with increased odds of being a smoker and exposure to secondhand smoke in China^11,12^. But the mechanism how cigarette gifting beliefs influence gifting and sharing behavior, and whether these gifting behaviors in turn promote smoking behaviors is unknown. Secondly, the Behavioral Ecological Model also highlighted cultural and social factors as important determinants of behavior^11,13,14^. Social participation is the process of socialization of individual behavior^15^. Individuals who are exposed to a wide range of social environments would learn from social norms. Social norms play a crucial role in individual behavior in China, where collectivist values are predominant^15^. According to Social Influence Theory and Behavioral Accessibility Theory, sharing and gifting cigarettes occurring during participation in all kinds of social activities may increase contact with cigarette smoking for both non-smokers and smokers attempting to quit^15^. That is why both the domestic and transnational tobacco industries promote the cigarette gifting custom in China as an essential strategy to expand tobacco marketing^4,16^.

Given the fundamental differences in political, economic, societal and cultural concerns between countries, tobacco control efforts and evidence-based experience generated from foreign studies cannot be directly applicable to the situation in China. Cigarette sharing and gifting plays an important role in boosting the smoking epidemic in China. However, few studies have empirically tested the smoking behavioral outcomes attributable to cigarette gifting and sharing^11^. Furthermore, these existing studies were restricted to qualitative studies with small sample sizes, and far from reflecting the cultural variability across different regions of China^5,7,17,18^. In order to address the research gap in the existing literature on the phenomenon of cigarette sharing and gifting, quantitative studies with large sample data at the regional/provincial level are necessary to support evidence-based tobacco control practices for the consideration of cigarette gifting culture in China. The purpose of this study is to portray the patterns and prevalence of cigarette sharing and gifting, and to identify their associated factors across different regions of China, and to explore the effect of these social practices on smoking.

## METHODS

### Study design and participants

The data for this cross-sectional study come from sampling the heads of households (HH) in two provinces of China in 2020. This study utilized a multistage sampling design in the survey. In Stage 1, one university each from Guangdong and Shaanxi Province was selected based on their diverse regional characteristics and existing research collaboration with the primary investigators. Guangdong is a highly developed coastal province in southern China with a population of 126.24 million and US$12789 per capita GDP. In contrast, Shaanxi is a northwestern inland province with 39.55 million people and US$9611 per capita GDP^19^. In Stage 2, the sampling strategy involved the selection of classes within each university. All classes that had health professional courses were selected from each university. The total eligible population of the students from these two universities were 2048 in Guangdong province and 921 in Shaanxi province. In Stage 3, the students who enrolled in the chosen classes and also came from the same province where the university was located were invited to distribute questionnaires and collect data from their heads of the household. Out-of-province students were excluded from this study because they might have been less successful in recruiting their heads of the households, thus introducing bias if they were invited to participate. In Stage 4, eligible students were encouraged to distribute the survey link to their parents. A total of 1240 HHs in Guangdong Province and 755 HHs in Shaanxi Province were recruited. The inclusion criteria for survey participants were the self-identified household head of the family who was both born and still living in the same local province in the past 12 months. Finally, a valid sample of 982 from Guangdong Province and 530 from Shaanxi Province were finalized for the data analysis. Our survey was developed on the Wenjuanxing Platform (https://www.wjx.cn/app/survey.aspx) and conducted online from 30 April to 30 July 2020. Given that survey period was during the early stages of the COVID-19 outbreak, all students were required to take online courses at home where they could contact their parents directly. The investigators were the aforementioned eligible students, who had received two-hour online training on the survey content and procedures. Each participant had an opportunity to seek information to be clarified about the questionnaire items from their college-attending children at home.

### Measures

*Sociodemographic characteristics*

The following sociodemographic information was collected during the survey: date of birth, gender, place of birth, place of residence, place of growing up before the age of 13 years, ethnicity, marital status, educational level, occupation, and per capita annual family income.

*Smoking status*

Respondents were asked whether they currently smoked; response options were: ‘Yes, smoke every day’, Yes, smoke on one or more days but not every day’, and ‘No’^20^. Respondents who smoked every day were classified as daily smokers; those who smoked on one or more days but not every day were classified as occasional smokers, and those who did not smoke were classified as non-smokers. Occasional smokers were combined with daily smokers to form a dichotomous indicator for smoking status.

*Sharing cigarette behavior*

Sharing cigarettes was defined as offering and accepting of single cigarettes^21,22^. Participants were asked about their personal cigarette sharing behavior over the last 12 months, including whether they had offered a single cigarette to others or received a single cigarette from others, who they had offered to, for what reasons they shared cigarettes, and in which situations the shared cigarettes were received.

*Gifting cigarette behavior*

Gifting cigarettes was defined as giving and receiving at least one unopened pack of cigarettes^21,22^. Participants were asked about their personal cigarette gifting behavior over the last 12 months, including whether they had offered gift cigarettes to others or received gift cigarettes from others, for what purpose they offered, for what reasons they took cigarettes as a gift, and in which situations the gift cigarettes were received.

*Beliefs about cigarette gifting*

Eight items for beliefs about cigarette gifting were assessed, including viewpoints about the functions, price perception and limitations, warning labels, and health hazards of gifting cigarettes. Items were rated on a 5-point Likert type scale, and ranged from 1 (strongly disagree) to 5 (strongly agree). Item scores were summed to attain a total belief score which ranged from a minimum value of 8 to a maximum value of 40. The higher the total score, the greater the agreement on gifting cigarettes. Cronbach’s alpha coefficient for beliefs toward gifting cigarettes was 0.81, which suggested a good reliability of this scale. Following prior practice, a score above the mean signified a high score for beliefs.

*Social participation*

Social participation was the scaled monthly frequency of ‘meeting with others for food and drink’, ‘going to leisure activities organized by your local work unit or commune’, ‘volunteering for public causes’, and ‘meeting with family members for entertainment’^23,24^. The frequency of these collective activities was rated as: never, once a few months, once a month, 2–3 times per month, and ≥4 times per month. A total social participation score was derived by summing item scores. The higher the score, the greater the participation in social activities. Cronbach’s alpha coefficient for social participation was 0.81 suggesting good reliability.

### Statistical analysis

All survey data were entered into a Microsoft Excel database, and then imported into SPSS (version 22.0) for statistical analysis. Descriptive statistics were calculated for sociodemographic variables, the patterns of cigarette sharing and gifting behaviors, and their beliefs about cigarette gifting. Chi-squared analyses were conducted to determine differences in both offering and receiving the shared and gifted cigarette across sociodemographic characteristics, beliefs, and smoking status. The independent variables in the logistic regression analysis were those variables that emerged as statistically significant for cigarette sharing and gifting behaviors in the chi-squared tests. A Wald test was used to test the statistical significance of each coefficient in the model. The odds ratio (OR) expresses the relative likelihood of having a probable behavior for offering and receiving the shared and gift cigarettes. We also constructed five models for logistic regression analyses of smoking status to test the role of sharing and gifting cigarettes. All models included statistically significant sociodemographic variables and gifting beliefs. Four categories of sharing and gifting behaviors were entered into Models 1 through 4, respectively. In the full model, four categories of social exchange behaviors of cigarettes were examined simultaneously.

## RESULTS

### Individual sociodemographic characteristics and smoking behavior

The average age of the participants was 47.8 (SD: 9.3) years. The male household head accounted for 82.5%, while the female household head accounted for 17.5%. The majority of participants were Han ethnic (99.3%) and married (88.2%). A sociodemographic profile is presented in Table 1. Forty-two percent of participants (n=634) reported being current smokers, of which 33.1% were daily smokers, and 8.8% were occasional smokers.

**Table 1:** The distribution, n (%), of cigarette sharing and gifting across demographic characteristics, China 2020 (N=1512)

*Variables*	*Total n (%)*	*Sharing cigarettes*		*Gifting cigarettes*	
		*Offering*	*Receiving*	*Offering*	*Receiving*
**Total**		518 (34.3)	841 (55.6)	361 (23.9)	280 (18.5)
**Age** (years)		p=0.099	p=0.031*	p=0.634	p=0.158
<45	336 (22.2)	107 (31.8)	171 (50.9)	74 (22.0)	56 (16.7)
45–49	593 (39.2)	192 (32.4)	323 (54.5)	147 (24.8)	102 (17.2)
≥50	583 (38.6)	219 (37.6)	347 (59.5)	140 (24.0)	122 (20.9)
**Gender**		p<0.001**	p<0.001**	p<0.001**	p<0.001**
Male	1248 (82.5)	509 (40.8)	818 (65.5)	331 (26.5)	266 (21.3)
Female	264 (17.5)	9 (3.4)	23 (8.7)	30 (11.4)	14 (5.3)
**Ethnicity**		p=0.340	p=0.004**	p=0.076	p=0.130
Han	1502 (99.3)	516 (34.4)	849 (55.9)	361 (24.0)	280 (18.6)
Minority	10 (0.7)	2 (20.0)	1 (10.0)	0 (0)	0 (0)
**Marital status**		p=0.004**	p<0.001**	p=0.001**	p=0.001**
Married	1334 (88.2)	474 (35.5)	774 (58.0)	337 (25.3)	263 (19.7)
Others	178 (11.8)	44 (24.7)	67 (37.6)	24 (13.5)	17 (9.6)
**Place of residence**		p=0.288	p<0.001**	p=0.591	p=0.406
Rural area	715 (47.3)	257 (35.9)	431 (60.3)	173 (24.2)	142 (19.9)
Micropolis	437 (28.9)	149 (34.1)	234 (53.5)	109 (24.9)	78 (17.8)
Large- and medium-sized cities	360 (23.8)	112 (31.1)	176 (48.9)	79 (21.9)	60 (16.7)
**Place of growing up before 13 years**		p=0.319	p=0.004**	p=0.695	p=0.200
Rural area	1296 (85.7)	447 (34.5)	733 (56.6)	305 (23.5)	240 (18.5)
Micropolis	147 (9.7)	53 (36.1)	83 (56.5)	37 (25.2)	32 (21.8)
Large- and medium-sized cities	69 (4.6)	18 (26.1)	25 (36.2)	19 (27.5)	8 (11.6)
**Education level**		p=0.081	p=0.018*	p=0.069	p=0.300
Elementary school or less	282 (18.7)	102 (36.2)	163 (57.8)	66 (23.4)	48 (17.0)
Junior high school	595 (39.4)	213 (35.8)	344 (57.8)	161 (27.1)	124 (20.8)
High school	353 (23.3)	125 (35.4)	201 (56.9)	80 (22.7)	62 (17.6)
Junior college, college or higher	282 (18.7)	78 (27.7)	133 (47.2)	54 (19.1)	46 (16.3)
**Occupation**		p=0.015*	p=0.006**	p=0.194	p=0.053
Manager/owner	31 (2.1)	14 (45.2)	18 (58.1)	11 (35.5)	9 (29.0)
White-collar	244 (16.1)	72 (29.5)	128 (52.5)	50 (20.5)	42 (17.2)
Blue-collar	670 (44.3)	249 (37.2)	402 (60.0)	162 (24.2)	133 (19.9)
Service class	229 (15.1)	86 (37.6)	131 (57.2)	63 (27.5)	49 (21.4)
Irregular employment	338 (22.4)	97 (37.0)	162 (47.9)	75 (22.2)	47 (13.9)
**Annual household income** (RMB)		p=0.015*	p=0.015*	p=0.134	p=0.207
<20000	494 (32.7)	176 (35.6)	276 (55.9)	125 (25.3)	84 (17.0)
20000–49999	479 (31.7)	178 (37.2)	289 (60.3)	121 (25.3)	103 (21.5)
50000–79999	208 (13.8)	53 (25.5)	97 (46.6)	43 (20.7)	34 (16.3)
80000–99999	122 (8.1)	48 (39.3)	70 (57.4)	34 (27.9)	26 (21.3)
≥100000	209 (13.8)	63 (30.1)	109 (52.2)	38 (18.2)	33 (15.8)
**Province**		p<0.001**	p<0.001**	p<0.001**	p<0.001**
Guangdong	982 (64.9)	258 (26.3)	489 (49.8)	175 (17.5)	155 (15.8)
Shaanxi	530 (35.1)	260 (49.1)	352 (66.4)	189 (35.7)	125 (23.6)
**Smoking status**		p<0.001**	p<0.001**	p<0.001**	p<0.001**
Daily smoker	501 (33.1)	372 (74.3)	418 (83.4)	199 (39.7)	180 (35.9)
Occasional smoker	133 (8.8)	85 (63.9)	106 (79.7)	54 (40.6)	45 (33.8)
Non-smoker	878 (58.1)	61 (6.9)	317 (36.1)	108 (12.3)	55 (6.3)
**Social participation^a^**		p=0.875	P=0.075	p=0.042*	p=0.003**
High score	696 (46.0)	237 (34.1)	370 (53.2)	183 (26.3)	151 (21.7)
Low score	816 (54.0)	281 (34.4)	471 (57.7)	178 (21.8)	129 (15.8)
**Cigarette gifting beliefs^a^**		p<0.001**	p<0.001**	p<0.001**	p<0.001**
High score	508 (33.6)	240 (47.2)	341 (67.1)	175 (34.4)	151 (21.7)
Low score	1004 (66.4)	278 (27.7)	500 (49.8)	186 (18.5)	129 (15.8)

a The cutoff value is the mean score. The average item score was higher than the mean score, indicating a high score for social participation and cigarette gifting beliefs. RMB: 1000 Chinese Renminbi about US$160.

* p<0.05;

** p<0.01.

### The beliefs about cigarette gifting

Table 2 shows the beliefs towards gifting cigarettes. The total score for the belief scale was 23.03 (SD: 5.16), and the mean item score was 2.88 (SD: 0.65). A considerable proportion of participants agreed that gifting cigarettes helps them maintain relationships (38.6%) and solve practical problems (23.9%). More than one-quarter of respondents agreed that the more expensive the cigarette, the more suitable it is to give away, and if the price of cigarettes goes up, they will still buy and use them as gifts. Similarly, around one-quarter of people acknowledged that they would ignore the health hazards of cigarettes when giving cigarettes to others as gifts, and a clear picture of the warning label on the packaging would prevent them from gifting cigarettes to others. The score of each item of beliefs regarding cigarette gifting was significantly higher among smokers than non-smokers, except for beliefs about the graphic warning label for gifting (t= -3.26, p<0.01).

**Table 2:** Beliefs about cigarette gifting among the total sample across different smoking status groups, China 2020 (N=1512)

*Beliefs*	*All participants n (%)*			*Mean (SD)*		*t*	*p*
	*Strongly disagree/disagree*	*Neutral*	*Strongly agree/agree*	*Smokers*	*Non-smokers*		
1. The more expensive the cigarette, the more suitable to give away	373 (24.7)	703 (46.5)	436 (28.8)	3.19 (0.98)	2.92 (0.98)	5.28	<0.001**
2. Gifting cigarettes helps me maintain relationships	306 (20.2)	622 (41.1)	584 (38.6)	3.45 (0.91)	2.97 (1.01)	9.58	<0.001**
3. Gifting cigarettes helps me solve practical problems	454 (30.0)	696 (46.0)	362 (23.9)	3.16 (0.99)	2.69 (0.98)	9.18	<0.001**
4. When gifting cigarettes to others, I don’t think about the health hazards of the cigarettes	452 (29.9)	678 (44.8)	382 (25.3)	3.15 (0.99)	2.69 (1.07)	8.91	<0.001**
5. I wouldn’t buy cigarettes and gift them if they have a clear picture of a tobacco warning on the package	392 (25.9)	727 (48.1)	393 (26.0)	2.92 (0.96)	3.08 (0.99)	-3.26	0.001**
6. If the price of cigarettes goes up, I’ll still buy and use them as gifts	384 (25.4)	734 (48.5)	394 (26.1)	3.21 (0.92)	2.77 (0.95)	8.98	<0.001**
7. The price-limit policy limits my choice of cigarettes as expensive gifts	416 (27.5)	785 (51.9)	311 (20.6)	2.96 (0.95)	2.79 (0.92)	3.52	<0.001**
8. If there is a channel in the market to buy cigarettes that are more expensive than 1000 RMB a carton, I am very willing to buy them as gifts	877 (58.0)	510 (33.7)	125 (8.3)	2.30 (1.04)	2.17 (1.01)	2.47	0.014*

* p<0.05;

** p<0.01.

### The patterns of sharing cigarette behavior

Among the participants, 34.3% (95% CI: 31.9–36.7) offered a single cigarette to others, while 55.6% (95% CI: 53.1–58.1) reported they had received a single cigarette in the past twelve months. The most frequent recipients of shared cigarettes were friends (87.6%), colleagues (69.1%), relatives (68.7%), and leaders/clients (45.6%). About one-quarter of participants reported sharing individual cigarettes with their family members. The most common reasons for sharing cigarettes were for basic meeting etiquette (80.7%), welcoming guests and showing intimacy (79.2%), and social engagement for work (59.8%). Accordingly, most participants received the shared cigarette at friends/family gatherings (79.1%), festive occasions (67.4%), first time meeting (64.6%), and at work (57.7%).

### The patterns of gifting cigarette behavior

Among the participants, 23.9% (95% CI: 21.7–26.0) offered gift cigarettes to others, while 18.5% (95% CI: 16.6–20.5) reported they received gift cigarettes in the past twelve months. The results showed that the majority of participants offered packaged cigarettes as gifts to their friends (67.6%), relatives (67.3%), leaders/clients (54.3%), and colleagues (46.5%). Approximately one-third of participants still sent gift cigarettes to their family members. The purposes for offering packaged cigarettes were sending a gift to others (79.5%), working demands (65.4%), or payment of remuneration (21.6%). The most common reasons the participants took the cigarettes as gifts were convenience of choice (57.3%) and the nature of clear value (54.8%). Holidays such as Spring Festival, weddings, or other essential days were the most prevalent situations where participants received gift cigarettes.

### The associated factors with cigarette sharing and gifting

The results from the chi-squared tests in Table 1 demonstrate that gender, marital status, region, smoking status, and cigarette gifting beliefs were all significantly associated with both offering and receiving shared and gift cigarettes. While all demographic variables were associated with receiving shared cigarettes except for social participation, occupation and annual household income were associated with offering shared cigarettes as well. Social participation was associated with both offering and receiving gift cigarettes.

The results from the multiple logistic regression analysis in Table 3 further showed that the male household heads were 4.93 (95% CI: 2.36–10.32) times more likely to offer a shared cigarette and 10.49 (95% CI: 6.60–16.68) times more likely to receive a single cigarette than the female. People with Han ethnicity were 11.55 (95% CI: 1.14–116.68) times more likely to receive a shared cigarette than those of minority ethnicity. Married people were more likely to receive shared cigarettes (AOR=1.55; 95% CI: 1.02–2.37) and gift cigarettes (AOR=2.14; 95% CI: 1.49–3.90), and 2.41 (95% CI: 1.49–3.90) times more likely to offer gift cigarettes than those who were unmarried. Participants from Shaanxi Province had a higher likelihood of offering shared cigarettes (AOR=2.18; 95% CI: 1.59–2.99) and gift cigarettes (AOR=2.13; 95% CI: 1.63–2.77) than people from Guangdong province. People who had a higher frequency of social participation were 1.32 (95% CI: 1.02–1.71) times and 1.59 (95% CI: 1.19–2.11) times more likely to offer and receive gift cigarettes, respectively. Cigarette gifting beliefs and smoking status were prominent predictors for sharing and gifting cigarettes. People who had high cigarette gifting beliefs, or were daily smokers and occasional smokers, were more likely to both offer and receive the gifted and shared cigarettes. Those who were daily smokers were 29.05 (95% CI: 20.57–41.03) times more likely to share cigarettes than those who were non-smokers.

**Table 3:** Logistic regression results of sociodemographic factors associated with cigarette sharing and gifting among all participants, China 2020 (N=1512)

	*Sharing cigarettes AOR (95% CI) ^a^*		*Gifting cigarettes AOR (95% CI) ^a^*	
	*Offering*	*Receiving*	*Offering*	*Receiving*
**Gender**				
Male	4.93 (2.36–10.32)**	10.49 (6.60–16.68)**		
Female	1	1		
**Ethnicity**				
Han		11.55 (1.14–116.68)*		
Minority		1		
**Marital status**				
Married		1.55 (1.02–2.37)*	2.41 (1.49–3.90)**	2.14 (1.23–3.71)**
Others		1	1	1
**Annual household income** (RMB)				
<20000	1.13 (0.70–1.84)			
20000–49999	1.09 (0.67–1.76)			
50000–79999	0.69 (0.39–1.22)			
80000–99999	1.99 (1.02–3.92)*			
≥100000	1			
**Province**				
Shaanxi	2.18 (1.59–2.99)**	1.48 (1.13–1.93)**	2.13 (1.63–2.77)**	
Guangdong	1	1	1	
**Smoking status**				
Daily smoker	29.05 (20.57–41.03)**	5.26 (3.94–7.02)**	3.94 (2.98–5.20)**	7.73 (5.52–10.81)**
Occasional smoker	16.69 (10.52–26.50)**	4.68 (2.89–7.57)**	3.69 (2.43–5.60)**	6.98 (4.41–11.03)**
Non-smoker	1	1	1	1
**Social participation^b^**				
High score			1.32 (1.02–1.71)*	1.59 (1.19–2.11)**
Low score			1	1
**Cigarette gifting beliefs^b^**				
High score	1.41 (1.03–1.93)*	1.53 (1.17–2.01)**	1.60 (1.23–2.09)**	1.68 (1.26–2.24)**
Low score	1	1	1	1

a The statistically significant variables from univariate analysis in Table 1 were included in each model, correspondingly. AOR: adjusted odds ratio.

b The cutoff value is the mean score. The average item score was higher than the mean score, indicating a high score for social participation and cigarette gifting beliefs. RMB: 1000 Chinese Renminbi about US$160.

* p<0.05;

** p<0.01.

### Smoking outcome predicted by gifting beliefs and behaviors

The results from Table 4 demonstrate that both offering and receiving shared or gift cigarettes, and gifting beliefs were all associated with smoking. However, when those four categories of behaviors were accounted for in the full model simultaneously, the effect of offering gift cigarettes was found to be not significant. The household heads who offered shared cigarettes, or received shared and gift cigarettes, were 19.17 (95% CI: 13.31–27.61), 1.50 (95% CI: 1.08–2.09) and 2.58 (95% CI: 1.66–4.00) times more likely to be current smokers, respectively. The participants who had a higher score of belief about cigarette gifting had a 1.63 (95% CI: 1.20–2.22) times higher likelihood of being a smoker.

**Table 4:** Logistic regression analysis for predicting smoking status by behaviors and beliefs towards cigarette sharing and gifting among all participants, China 2020 (N=1512)

	*Model 1^b^ AOR (95% CI) ^a^*	*Model 2^b^ AOR (95% CI) ^a^*	*Model 3^b^ AOR (95% CI) ^a^*	*Model 4^b^ AOR (95% CI) ^a^*	*Full model ^c^ AOR (95% CI) ^a^*
**Cigarette sharing behaviors**					
**Offering**					
Yes	25.95 (18.81–35.80)**				19.17 (13.31–27.61)**
No	1				1
**Receiving**					
Yes		4.98 (3.82–6.49)**			1.50 (1.08–2.09)*
No		1			1
**Cigarette gifting behaviors**					
**Offering**					
Yes			3.64 (2.74–4.84)**		0.78 (0.52–1.19)
No			1		1
**Receiving**					
Yes				6.70 (4.76–9.44)**	2.58 (1.66–4.00)**
No				1	1
**Cigarette gifting beliefs**					
High score	1.76 (1.30–2.38)**	1.84 (1.43–2.38)**	1.85 (1.44–2.38)**	1.83 (1.41–2.36)**	1.63 (1.20–2.22)**
Low score	1	1	1	1	1

a AOR: adjusted odds ratio; the statistically significant sociodemographic characteristics for smoking such as gender, education level and region were adjusted.

b Four categories of cigarette exchange behaviors were entered into Model 1 through Model 4, respectively.

c Four categories of cigarette exchange behaviors were entered into the full model, simultaneously.

* p<0.05;

** p<0.01.

## DISCUSSION

This study depicts the phenomenon of cigarette sharing and gifting comprehensively, distinguishing the difference between actively offering and passively receiving behaviors. The prevalence of cigarette sharing and gifting in the current study was lower than that from a nationwide survey administered between 2017 and 2018^7^, but higher than that in research on tobacco utilization conducted in 2005, which indicated that over 23% of the smokers had either received or given cigarettes as gifts^9^. In addition, the International Tobacco Control (ITC) China Survey in 2007 revealed that the incidence of receiving cigarettes as a gift from the most recent cigarette acquisition was 3.5%^8^. Compared to previous studies, the distinction for inconsistent cigarette sharing and gifting prevalence might be induced by the different estimation methods, survey time, and targeting subjects. However, these data supported the assumption that cigarette sharing and gifting were still endemic in China. More interestingly, it was found that more than one-third of non-smokers (36.9%) reported they had received a single cigarette, which was similar to the results from a prior study^7^. This was also consistent with the results from the pattern of sharing cigarettes for mutual corroboration. Four-fifths of respondents reported their motivation for sharing cigarettes was to demonstrate basic meeting etiquette, welcoming guests, and social intimacy. Sharing cigarettes is a pervasive Chinese behavior in which people offer individual cigarettes to others in specific social settings, which has become an invaluable avenue for social communication^21^. That might explain why non-smokers could receive a single cigarette. In some social situations, people share cigarettes regardless of whether the recipients smoke or not. It provides caution that sharing cigarettes with non-smokers might increase the risk of boosting smoking initiation or inducing relapse.

Regarding cigarette sharing and gifting patterns, the results showed that friends were the most likely recipients of both sharing cigarettes and gifting cigarettes. At the same time, more than two-thirds of participants reported they had similar practices to their relatives. Nearly one-third would even give cigarettes to family members as a gift. These data support the assertion that gift-giving behavior has a role in maintaining family and social relationships and building social status in China^4^. Notably, more than 91% of participants in this study reported receiving gift cigarettes during festival occasions, which have become the most common occurrence. Our current quantitative data confirmed previous viewpoints that gifting cigarettes often occurs during weddings or festivals of the Chinese New Year, which cater to the tobacco companies’ marketing strategies by connoting their brands into festival values such as happiness, warmth, friendship, and celebration^4,6^. Therefore, related tobacco control programs are urged to break social acceptance and change social norms toward gifting and sharing cigarettes.

Similar to the results from prior research^7^, our study also showed gender differences in the behaviors of sharing cigarettes, especially for receiving behaviors. One potential explanation may be the smoking prevalence of gender differences. Another possible explanation might be that social sharing is generally reserved for adult men and is often associated with the workplace and other professional interactions where women are by and large exempt from situations requiring cigarette sharing^21^. In line with the results generated by previous studies, married people were more likely to share and gift cigarettes than unmarried people^7^. Chinese cultural practices of family collectivism particularly reinforce the responsibility and duty of married couples during festival seasons, and gift exchanges have served important functional purposes in this regard.

The phenomenon of cigarette sharing and gifting in Shaanxi, one of the northwestern inland provinces of China, was found to be more widespread than it is in Guangdong, a highly developed coastal province in southern China. It is partially explained by the regional differences in smoking prevalence^25^. Besides, regional differences in economic development and social culture might contribute the substantial variation. Culturally, the north has been influenced by nomadic culture and the south by an agrarian one^26^. Generally, residents living in the south have benefited from the faster economic development. Economic imbalances and education inequality may reflect an individual’s health-related literacy and capacity to make health-conscious decisions such as tobacco use^14^. Meanwhile, the northern residents were deeply influenced by Confucianism which highlights etiquette. The practices for giving and receiving gifts to create relationships frequently occurs in the north of China^27^. Moreover, north-south regional and cultural differences in psychosocial characteristics may also partially explain this disparity^28^.

Smoking status was closely associated with cigarette sharing and gifting. From the viewpoint of Behavioral Susceptibility Theory, conducting a given behavior becomes convenient, and this behavior will gradually increase^23^. Smokers would bring their own cigarettes with them where cigarettes were available to share and gift to others. Additionally, smokers tend to smoke cigarettes, especially prestigious ones with displays of social and cultural values of respect and personal honor^4^. Sharing and gifting cigarettes has been an indispensable way for smokers to gain social approbation and build and maintain interpersonal relationships. Furthermore, smokers may be more receptive and enjoyable to the cigarette culture of courtesy reciprocity than non-smokers.

The present study found that beliefs about gifting cigarettes was associated with both cigarette sharing and gifting. Thus, our research supports the strong connection between beliefs and behaviors^7,23,29^. Moreover, smokers had a higher agreement on cigarette gifting than non-smokers, which in turn enhanced the relationship between smoking and cigarette gifting. Remarkably, the evidence that more than one-fifth of people still hold irrational beliefs about cigarette gifting, should be paid more attention by researchers and the authorities.

Another interesting finding in the current study disclosed that social participation was significantly associated with cigarette gifting, but not related to cigarette sharing. This finding implied that gift-giving behaviors might play a more critical role in social relationships than sharing behaviors. Meanwhile, the results align with the assumptions of the dark side of social participation, indicating that social participation in collective activities may unintentionally facilitate at-risk behaviors such as cigarette gifting behavior^30^.

Regarding behavioral outcomes of cigarette sharing and gifting behaviors, the current study demonstrated these practices were the key contributors to smoking, as implicitly suggested by a previous study^11^. It is noteworthy that the behavior of actively sharing cigarettes was significantly predicting smoking. Receiving a single cigarette or packs of cigarette gifts were both associated with increased odds of being a smoker, which confirmed and amplified the previous results from the male participants to general people including both genders^11^. The results from the current study also reflected that smoking socially is frequently occurring by exchanging cigarettes.

Overall, the results of this study provided some policy implications for intervention and prevention. Firstly, the tobacco control programs in the Chinese context should not neglect the culture of cigarette sharing and gifting when introducing the experience of foreign countries in tobacco control due to the relatively rare phenomenon of these social practices in other cultures. This study may also contribute a literature basis on substance sharing and gifting for some similar cultures of other countries. Secondly, given their potential side effects on smoking, the identification of specific factors associated with cigarette sharing and gifting is beneficial when aiming to understand the onset and course of sharing and gifting behaviors, and further for preventing increases in these behaviors that may subsequently lead to more disease burden and loss from smoking. Thirdly, recognizing the key contributors to these exchanging behaviors of cigarettes is a crucial step in enhancing the efficiency of tobacco control initiatives. The perspectives from specific populations such as male and married smokers in the northern region, individual social participation, and cigarette gifting beliefs should be incorporated into the intervention measures. These include advancing public and media advocacy for ‘refusing to offer a single cigarette to others’ or ‘say No to receiving cigarette gifts’ during social participation to break the cigarettes-social reinforcement link, and changing the image of cigarette packaging with graphic warnings to change social acceptance of cigarette gifts, starting with some northern pilot studies.

### Limitations

The cross-sectional design prohibits causal inferences. Recall bias and social desirability may occur by applying the self-report measures. This survey was administered online, and the survey link was delivered to parent respondents by students. The selection bias might influence the generalization and underestimate the prevalence of cigarette sharing and gifting because the sample only included the household heads whose children were college-attending students. Moreover, the bias of falsely responding by students themselves might exist, but logical checks from the data cleaning process have been conducted. It is also difficult to generalize the current findings, from data of two provinces, to the entire country.

## CONCLUSIONS

This study provides population-based estimates of the prevalence and associated factors of cigarette gifting and sharing utilizing a large sample size across different regions of China, with subdivisions of offering and receiving behaviors. Cigarette sharing and gifting were especially pervasive among male smokers and married people in Shaanxi Province. Beliefs about cigarette gifting and its impact on sharing and gifting behaviors were confirmed. Social participation was significantly associated with cigarette gifting. The potentially harmful behavioral outcomes attributable to cigarette gifting and sharing were also quantitatively assessed. Cigarette sharing behaviors and receiving gift cigarettes were found to predict tobacco use significantly. This study provides evidence-based data to support the design and implementation of pertinent tobacco control programs for the denormalization of the cigarette gifting and sharing culture.

## Data Availability

The data supporting this research are available from the authors on reasonable request.
